# Mind the gap between RNA and protein levels: A general phenomenon with implications for virology

**DOI:** 10.1371/journal.ppat.1014470

**Published:** 2026-07-30

**Authors:** Luca D. Bertzbach, Timon Kapischke, Lars Kaderali, Stephanie Pfaender, Wing-Hang Ip

**Affiliations:** 1 Department of Emerging Viruses, Leibniz Institute of Virology (LIV), Hamburg, Germany; 2 Institute of Bioinformatics, University Medicine Greifswald, Greifswald, Germany; 3 Department of Biology, University of Hamburg, Hamburg, Germany; Washington University School of Medicine in Saint Louis: Washington University in St Louis School of Medicine, UNITED STATES OF AMERICA

## Introduction

Accurate interpretation of gene expression during viral infection often relies on transcriptomic measurements, yet in both cell biology and virology it is now well established that RNA abundance is an imperfect proxy for protein levels. Early large-scale efforts to quantify gene expression, such as the landmark study by Schwanhäusser and colleagues, revealed that translation and protein degradation dominate steady-state protein abundance, sparking extensive debate and reanalysis [1]. Subsequent work refined these estimates, showing that while RNA contributes substantially, RNA-protein correlations are typically moderate and highly gene-specific, with transcript-protein relationships often showing only modest linear association (Pearson correlation coefficient *r* frequently around 0.5 or lower), meaning that mRNA levels only partially predict protein abundance [2–6]. Conceptual syntheses established that translation efficiency and protein stability are among the primary determinants of protein abundance, a view reinforced by gene-resolved analyses demonstrating that some transcripts closely track protein levels, whereas others are almost entirely decoupled [7].

These principles extend, and are frequently hijacked and amplified, during viral infection, enabling viruses to rewire host gene expression by decoupling RNA abundance from protein output to promote replication and evade immune detection. In virology, while transcriptional regulation sets the initial RNA pool, protein abundance is ultimately shaped by a cascade of post-transcriptional and post-translational mechanisms, including translation efficiency, RNA stability, translational control, and protein degradation, highlighting the nonlinear relationship between RNA and protein levels. Multi-omics and translational profiling studies across diverse viral systems consistently demonstrate that RNA abundance incompletely predicts protein output during infection. In human cytomegalovirus (HCMV) infection, quantitative temporal viromics reveals strong divergence between transcript and protein dynamics across both viral and host systems, with distinct temporal waves of protein expression that are only partially reflected at the RNA level, highlighting extensive post-transcriptional and post-translational regulation [8]. Extending this framework, transcriptomic analyses of HCMV gene expression during lytic and latent infection show that viral transcription is not governed by a simple linear cascade, but instead by multiple independently regulated gene modules. Rather than a strictly sequential program, viral genes are controlled through layered regulatory inputs, and latency is characterized by a distinct repression of immediate-early genes, redefining classical models of HCMV gene regulation and underscoring fundamentally different transcriptional control logic between infection states [9]. This layered regulatory logic, characterized by asynchronous activation and state-specific repression (e.g., of immediate-early genes during latency), results in nonuniform RNA accumulation, contributing to the poor correlation between RNA and protein levels even before translation or degradation is considered.

In SARS-CoV-2-infected cells, mapping of the viral RNA-protein interactome reveals that both viral and host RNAs form extensive, dynamic complexes with host RNA-binding proteins (RBPs) and viral proteins, collectively shaping the functional fate of transcripts. This indicates that RNA abundance alone is insufficient to predict or explain downstream functional protein-level outcomes, as functional interpretation depends on the specific RNA-protein complexes formed during infection [10]. Similarly, ribosome-associated translation remodeling during Dengue virus infection demonstrates that viral infection can selectively reprogram endoplasmic reticulum-associated translation machinery, leading to substantial discordance between RNA abundance and ribosome engagement, and therefore between transcript levels and protein synthesis output [11]. This decoupling is further reinforced by recent systems-level analyses of viral gene expression programs. In HIV-1 infection, global translational profiling shows that extensive RNA diversity generated through splicing and processing is subject to strong post-transcriptional filtering, such that only a subset of transcripts is productively translated into protein [12]. Likewise, during vaccinia virus infection, integrated proteomic and transcriptomic analyses reveal widespread gene-specific discordance between RNA and protein kinetics, underscoring dominant roles for translational control and protein stability in shaping the viral proteome [13].

Collectively, these observations argue that RNA-protein discordance is not experimental noise but a fundamental feature of gene expression regulation, driven by layered control at the levels of transcription, translation, and RNA/protein stability.

## 1. RNA abundance is only loosely predictive of viral protein levels

Transcriptomic data alone fail to reliably predict protein expression, as many genes exhibit low or no correlation, delayed responses, inverse relationships, or inconsistent coupling between RNA and protein levels [14]. Viral genes are particularly prone to decoupling due to temporal expression programs, polycistronic mRNAs, nested open reading frames, programmed ribosomal frameshifting, or compartmentalized replication. Alternative splicing and RNA modifications (such as m^6^A methylation) further diversify the transcriptome, generating multiple protein isoforms from a single gene or altering RNA stability, localization, and translation efficiency [15]. Translation of viral RNA is tightly coupled to, and often impeded by, RNA replication, and ribosomes can stall or displace replication complexes, creating a kinetic conflict that delays protein synthesis and contributes to RNA-protein discordance [16]. The distribution of RNA, translation, and protein abundance differs significantly, with replication compartments further distorting local RNA levels. Critically, assembled virions, usually containing one genome copy but numerous proteins, can accumulate intracellularly with long half-lives, skewing total RNA and protein measurements toward nonfunctional, stored material – such as mature virions that are not actively translating or replicating but persist in the cell. Thus, RNA-seq alone cannot define functional viral or host output [17]. The disconnect between RNA and protein levels is not merely a consequence of measurement artifacts, but is actively shaped: post-transcriptional mechanisms including microRNA-mediated silencing, RNA decay, and translational repression play a central role in shaping protein output [18]. Host cells deploy these pathways to target viral mRNAs for degradation or translational arrest, often through post-transcriptional gene silencing mediated by cellular or viral miRNAs. In parallel, RNA-protein complexes such as stress granules and P-bodies act as hubs for RNA storage, decay, and translational control, sequestering viral transcripts and limiting their expression [19,20]. Therefore, this discordance is not technical noise but reflects biological regulation, as demonstrated by multi-omics studies showing poor correlation between transcription and translation in infected cells.

## 2. Translation is a primary control point reshaped by infection

Viral infections frequently reprogram translation, inducing global host shutoff while selectively promoting viral mRNA translation via mechanisms such as cap-snatching, IRES-driven initiation, ribosome hijacking, or ribosomal pausing. These strategies allow viruses to prioritize their own protein synthesis despite host translational suppression. The ribosome, not the transcriptome, often determines infection phenotypes, as translation efficiency can override RNA abundance. For example, influenza virus uses cap-snatching to initiate viral mRNA translation, while other viruses exploit altered ribosome dynamics to favor viral over host protein production [21,22]. Notably, some viruses use specialized ribosomes (modified with unique ribosomal proteins or post-translational modifications) to translate viral mRNAs within virus-induced organelles [23]. Functional studies have shown that RPS25 is selectively recruited to the 40S ribosomal subunit during IRES-mediated initiation, enabling translation of viral mRNAs [24]. Viruses subvert these ribosomal features to enhance translation efficiency and evade host surveillance. This translational reprogramming underscores that protein levels are not dictated by RNA abundance alone, but by active, virus-driven control of the translation machinery.

## 3. Some viral life cycles intentionally decouple RNA and protein timing

Many viruses use temporal decoupling to optimize infection: early genes (e.g., regulatory proteins) are translated from low-abundance RNAs to generate potent protein bursts, while late structural genes accumulate RNA in advance, with translation delayed until assembly is ready. This allows for efficient coordination of replication and virion formation. For instance, herpesviruses employ multiple independently regulated temporal modules, with gene expression not strictly tied to classical transcriptional cascades [9]. In such cases, RNA abundance reflects “stored potential” rather than immediate function, highlighting that temporal regulation is a key viral strategy to maximize fitness and evade host defenses. Host cells deploy the nonsense-mediated decay (NMD) pathway as a frontline defense, targeting aberrant or foreign mRNAs for degradation. In response, viruses have evolved counterstrategies: they either induce NMD to eliminate host antiviral transcripts or modify their own RNAs to evade recognition, such as by altering 3′ UTRs or splicing patterns [25]. This ongoing molecular contest, where host surveillance shapes viral evolution and viral evasion reshapes host defenses, again demonstrates that RNA-protein discordance is not noise, but a functional signature of a dynamic host-pathogen interaction.

## 4. Post-translational regulation dominates protein abundance outcomes

Protein abundance is as much a product of degradation as of synthesis. Viruses actively manipulate host degradation pathways—degrading antiviral proteins while stabilizing viral proteins to prolong their functional lifespan. Host mRNA shutoff (e.g., via viral endonucleases or indirectly, by activation of cellular RNA degradation pathways) leads to rapid decline in host protein synthesis, while viral proteins may be stabilized through post-translational modifications or interactions with host factors [26]. For example, SUMOylation of viral proteins can alter their localization (and stability), with cascading effects on host proteostasis [27]. Accordingly, post-translational regulation, including targeted degradation, is a major driver of protein abundance changes during infection – and protein levels are not simply a reflection of transcription/RNA levels but a dynamic balance between synthesis and degradation.

## 5. RNA-protein discordance reflects biological regulation, not experimental noise!

Discrepancies between RNA and protein levels are not artifacts but meaningful regulatory features shaped by evolution, host defense, and viral countermeasures. They reflect temporal phase offsets, selective translation, and compartmentalization, strategies that optimize infection efficiency (while host cells attempt to counteract this by downregulating translation globally or targeting specific viral mRNAs for degradation, ultimately aiming to limit viral replication and spread). RBPs and noncoding RNAs (ncRNAs), for example, play a central role in this regulation, modulating RNA stability, localization, and translation in response to infection [28–30]. These mechanisms allow viruses to fine-tune protein expression without altering transcription, enabling rapid adaptation. Discordance is therefore not noise but a functional signature of infection, revealing layers of post-transcriptional control that are critical for viral fitness and host-pathogen interactions. Recognizing this is essential for interpreting omics data and understanding true biological function.

## Take home message

**Fig 1:**
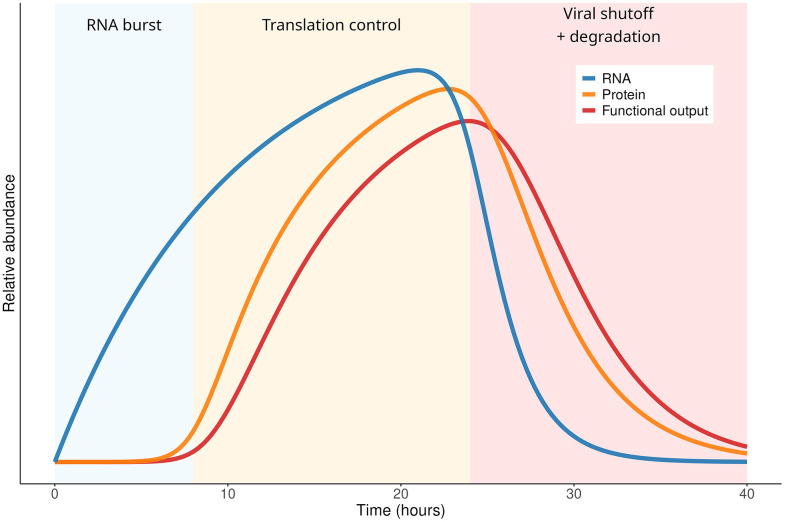
RNA vs protein trajectories during viral infection. Schematic and simplified time course of viral RNA, protein, and functional output (e.g., infectious particle production or reporter activity) following infection. All values are normalized to the maximum observed level for each component to enable direct comparison across molecules. RNA shows an early peak, reflecting rapid transcription. Protein accumulates with a delay, consistent with translation kinetics. The functional output generally closely parallels the protein curve, indicating that biological activity is limited by protein abundance and maturation rather than RNA levels. Importantly, assembled virions (containing one RNA copy but many proteins) may accumulate intracellularly and persist with long half-lives, contributing to elevated total protein and RNA signals without reflecting active gene expression. This can artificially inflate abundance measurements and obscure true regulatory dynamics. Thus, the strong correlation between protein and functional output, despite the RNA-protein gap, underscores the importance of measuring biologically active output rather than total abundance alone.

Taken together, RNA-protein discordance in general as well as in viral infection systems should not be viewed as a limitation of transcriptomics, but as a hallmark of multilayered post-transcriptional as well as post-translational regulation. While discrepancies between RNA and protein levels have historically been attributed to technical variability—such as differences in detection sensitivity or data analysis—recent multi-omics studies reveal that this divergence is largely biological, not technical. The growing body of evidence shows that transcript-protein uncoupling is widespread, driven by dynamic processes including transcriptional and translational control, selective protein degradation, viral hijacking of ribosomes, and the accumulation of nonfunctional, packaged viral components. These mechanisms (e.g., delayed translation of late viral genes, RBP and ncRNA-mediated RNA regulation, and compartmentalization of replication and translation) allow viruses to fine-tune protein output independently of RNA abundance (Fig 1, Table 1). Assembled virions, with their long half-lives and high protein-to-RNA ratios, further distort total abundance measurements, emphasizing that high RNA or protein levels do not equate to functional activity. Thus, RNA levels alone are insufficient to predict biological outcomes. Instead, *in silico* models should explicitly incorporate transcriptional, translational, post-transcriptional, and post-translational regulatory processes, while *in vitro* and *in vivo* studies should integrate transcriptomic measurements with direct assessments of protein abundance and function and move beyond transcript-centric biases. Rather than viewing RNA-protein discordance as noise across the continuum from gene induction to functional protein responses, it should be recognized as a key indicator of dynamic regulatory mechanisms including post-transcriptional and translational regulation, as well as protein stability. Understanding this gap is not about correcting data, but about uncovering the true process of infection, where biological function emerges not only from RNA abundance, but also from the precise spatiotemporal control of protein synthesis and stability.

**Table 1:** Key mechanisms of RNA-protein discordance in viral infection. Mechanisms are listed in the order they are discussed in the manuscript.

Mechanism	Biological role	Functional outcome
Temporal decoupling	Coordinates replication and virion assembly	Early proteins from low-abundance RNA; late proteins delayed
Programmed ribosomal frameshifting	Enables expression of multiple proteins from a single mRNA	Stoichiometric control of viral enzymes
Alternative splicing and RNA modifications	Diversifies transcriptome and regulates RNA stability and translation	Multiple isoforms; immune evasion
Compartmentalization of replication and translation*	Separates RNA synthesis from protein production	Local RNA accumulation without immediate translation
Ribosome specialization and IRES-driven initiation	Prioritizes viral translation during host shutoff	Selective viral translation despite host shutoff
Nonsense-mediated decay (NMD) manipulation	Host defense vs. viral evasion	Degrades host antiviral transcripts; protects viral RNAs
Post-translational regulation (PTMs, degradation)	Controls protein stability, localization, and function	Prolonged viral protein lifespan; host factor degradation
RNA-protein interactomes and granules	Regulates RNA fate and translation efficiency	Sequestration in stress granules; selective translation
RBP and ncRNA-mediated RNA regulation	Modulates RNA stability, localization, and translation	Fine-tuning of viral gene expression

*Note: Compartmentalization creates spatial decoupling, allowing RNA accumulation without immediate translation, contributing to RNA-protein discordance.
